# Supplementary material for: Methylated CpG ODNs from Bifidobacterium longum subsp. infantis Modulate Treg Induction and Suppress Allergic Response in a Murine Model
Source: Int J Mol Sci. 2025 Jul 14;26(14):6755. doi: 10.3390/ijms26146755 (PMC12295409; doi:10.3390/ijms26146755)
Supplement: Supplementary file 1 [file ijms-26-06755-s001.zip › ijms-3736943-supplementary.pdf]

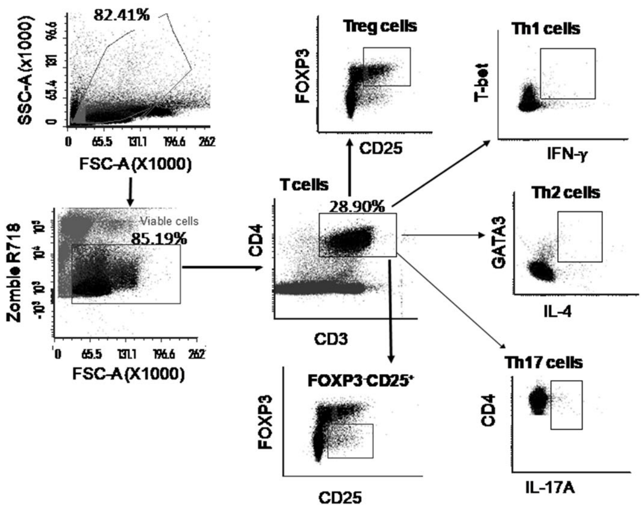

Figure S1. Schematic gate strategy: murine splenic mononuclear cells were staining with Zombie 718 (1:500) to assess cell viability, followed by extracellular and intracellular staining. Single cells were identified by plotting forward scatter width (FSC-W) against side scatter area (SSC-A). Live cells were gated as Zombie-negative. T cells were identified as $CD3^{+}CD4^{+}$ cells within the live cells. Regulatory T cells (Tregs) were gated as $CD4^{+}CD25^{+}FOXP3^{+}$ cells. Th1 and Th2 subsets were distinguished using specific fluorescent markers: Th1 cells as $T\text{-}bet^{+}$ $IFN\text{-}\gamma^{+}$ and Th2 cells as $GATA3^{+}IL\text{-}4^{+}$. Th17 cells were gated based on IL-17A expression. Live/dead dye and fluorescence-minus-one (FMO) controls were used for all antibodies except one, to validate the gating strategy for each population. Compensation was performed using Invitrogen bead.
